# Stress Urinary Incontinence as a Chronic Stress Model: Clinical Evidence and Emerging Epigenetic Implications for Mental Disorders

**DOI:** 10.3390/biomedicines14092075

**Published:** 2026-09-15

**Authors:** Despoina Drivakou, Nikolaos Roussos, Iakovos Theodoulidis, Konstantinos Dinas, Themistoklis Mikos, Mary H. Kosmidis

**Affiliations:** 1 Urogynecology Unit, 2nd Department of Obstetrics & Gynecology, Aristotle University of Thessaloniki, Hippokration General Hospital, 546 42 Thessaloniki, Greece; 2 Urogynecology Unit, 1st Department of Obstetrics & Gynecology, Aristotle University of Thessaloniki, Papageorgiou General Hospital, 564 29 Thessaloniki, Greece; 3 School of Psychology, Lab of Neuropsychology & Behavioral Neuroscience, Aristotle University of Thessaloniki, 541 24 Thessaloniki, Greece; kosmidis@psy.auth.gr

**Keywords:** stress urinary incontinence, epigenetics, NR3C1, FKBP5, HPA axis, depression, chronic stress, translational psychiatry

## Abstract

Stress urinary incontinence (SUI) is conventionally viewed as a peripheral mechanical disorder, yet its psychological burden suggests that a broader biopsychosocial interpretation may be warranted. This perspective considers whether recurrent leakage, anticipatory anxiety, behavioural avoidance, and social evaluative threat may allow SUI to function as a chronic psychosocial stressor. It integrates clinical observations with established concepts in stress physiology, hypothalamic–pituitary–adrenal (HPA) axis regulation, allostatic load, inflammation, and stress-related epigenetic mechanisms, including FKBP5 and NR3C1. Direct molecular evidence in SUI populations remains limited; therefore, the proposed links are presented as a hypothesis-generating framework rather than as established causal pathways. The framework further emphasises the heterogeneity of patient outcomes. Differences in perceived symptom burden, coping, stress responsivity, prior vulnerability, and resilience may help explain why psychological symptoms improve after treatment in some individuals yet persist in others. These interacting psychosocial and biological processes may reinforce a feedback loop between symptom-related threat, sustained stress activation, and affective vulnerability. By moving beyond a solely mechanical model, this perspective supports a more interdisciplinary approach combining urogynecological and psychological assessment. The proposed stress-epigenetic framework is intended to guide future longitudinal and biomarker-informed research and to encourage more integrated approaches to the prevention and management of psychological burden in SUI.

## 1. Introduction

### 1.1. Overview of Stress Urinary Incontinence

Stress urinary incontinence (SUI) is a common pelvic floor disorder which is characterised by the involuntary leakage of urine when a person is engaged in activities that can increase pressure on the intra-abdominal region, such as sneezing, coughing, laughing, or physical exertion. It represents the most prevalent subtype of urinary incontinence among women and is reported to affect somewhere between 10 and 40% of adult females; this prevalence increases with parity, age, and menopausal status [1]. Even though SUI is not associated with direct mortality, its critical importance lies in its undeniable impact on overall well-being and functional capacity.

The pathophysiology of SUI is mostly attributed to the dysfunction of the pelvic floor musculature and impaired urethral support, which is a result of trauma related to childbirth, neuromuscular weakening, or connective tissue changes [2]. Standard management strategies include conservative interventions such as various types of muscle training for the pelvic floor and complex surgical procedures, specifically mid-urethral sling techniques, which can demonstrate a high rate of success in restoring continence.

Even after such advances, SUI cannot be understood fully as a purely mechanical issue. This condition is incorporated within the broader context of biopsychosocial well-being, where the symptoms experienced extend beyond the physiological dysfunction. Increasing focus has thereby been directed towards comprehending the multidimensional burden of SUI, specifically its social and psychosocial implications.

### 1.2. Psychosocial Burden and Quality of Life Impact

The impacts of SUI extend beyond its physical symptoms, resulting in a significant psychosocial effect that adversely impacts the quality of life of the patient. Women who have SUI report reduced self-esteem, embarrassment, and social withdrawal because of the fear of leakage and the perceived stigma attached to it [3]. Daily activities, which include occupational function, exercise, and intimate relationships, can be restricted and this can contribute to a reduced sense of well-being and autonomy. More importantly, the different experiences of symptoms can often outweigh the objective severity in determining the impairment in the quality of life. This has highlighted the main role of social and psychological factors in shaping the experience of SUI, thereby reinforcing the requirement for a broader conceptual framework.

Recent population-based studies have reported that women with SUI have experienced substantial impairments throughout multiple quality of life domains, which include social participation, occupational productivity, intimate relationships, and physical activity. High-impact epidemiological evidence has also shown that the burden of severe symptoms is related to significantly lower health-related and mental health quality of life scores in comparison to continental populations [4]. In many cases, patients have reported avoiding exercise, modifying daily routines, and restricting social engagement because of public embarrassment and fear of leakage. These findings have indicated that the burden of SUI has extended beyond physical inconvenience and has also affected broader psychosocial functioning, along with life participation.

### 1.3. Link Between SUI and Mental Health Outcomes

A large body of clinical and epidemiological research has stated that there is a strong relationship between adverse mental health outcomes and stress urinary incontinence. These adverse mental health outcomes are generally related to anxiety and depression. Various cross-sectional studies have consistently shown a high rate of prevalence of depressive symptoms among women who have SUI, compared with control groups, and the severity has often correlated with the burden of symptoms and perceived stress. Recent systematic evidence has also reported that women with urinary incontinence experienced nearly two-times-higher rates of depressive symptomatology in comparison to continent controls [5]. Longitudinal evidence has also suggested that this kind of relationship might be bidirectional: psychological distress can be a result of urinary symptoms and it can contribute towards them as well [6].

A number of mechanisms have been recommended for explaining this relationship. The unsettling unpredictability of leakage, the perceived loss of bodily control, and the fear of public embarrassment can contribute to persistent emotional strain and anticipatory anxiety [7]. Over time, these factors lead to maladaptive coping behaviours, which include social isolation and avoidance, which are established risk factors for depressive symptomatology.

Most importantly, while conservative and surgical treatments can improve continence, a clinically important subset of patients have continued to experience psychological distress even after the successful resolution of symptoms [8]. This kind of dissociation between the physical recovery of the patient and their emotional outcomes has suggested that there is an underlying mechanism which might extend beyond the physiological dysfunction, warranting a thorough exploration within a stress-based framework.

These findings have exposed an important limitation within conventional biomedical models of SUI, which have primarily interpreted the condition through peripheral mechanical dysfunction. Even though such models have explained continence failure effectively, they have not sufficiently accounted for persistent psychological distress, continued affective symptoms, or anticipatory anxiety after successful treatment. This theoretical gap supports the requirement for a broader stress-oriented framework [9].

### 1.4. Rationale for a Stress-Based Conceptualisation

Traditional biochemical models have conceptualised SUI primarily as a localised mechanical disorder which is centred on pelvic floor dysfunction and urethral support failure. Even though these models have explained the physiological basis of continence impairment, they have accounted inadequately for the persistent psychological burden, behavioural avoidance, anticipatory anxiety, and residual affective symptoms which are observed in many patients even after successful treatment [10]. This conceptual limitation indicates that SUI cannot be comprehended only through peripheral pathology and instead it might require a much broader framework which is capable of integrating sustained psychosocial stress with biological stress regulation systems.

### 1.5. Aim and Scope of the Perspective

This perspective integrates interdisciplinary evidence linking SUI with psychological distress, chronic stress physiology, and emerging epigenetic mechanisms. It asks whether SUI may be conceptualised as a chronic psychosocial stressor and considers how sustained symptom-related stress could interact with neuroendocrine and molecular pathways implicated in affective disorders. The proposed framework is situated within translational psychiatry. It is hypothesis-generating: it does not establish causality, but identifies plausible mechanisms and priorities for future empirical research.

## 2. Psychological and Affective Outcomes in Stress Urinary Incontinence

### 2.1. Epidemiology of Depression in SUI

Epidemiological evidence published in the past five years has demonstrated a consistently strong and clinically important link between SUI and depressive symptomatology. Meta-analyses and systematic reviews have indicated that women who have urinary incontinence have exhibited alarmingly higher rates of depression compared to continent populations; this type of relationship has persisted throughout diverse demographic and geographical contexts. Population-based studies have also suggested that this kind of linkage is not minor, but rather reflects a coherent comorbidity which can contribute to the overall burden of disease [11].

Large-scale observational studies have also reinforced such findings. As an example, community-based research has demonstrated that urinary incontinence is significantly linked with increased levels of stress, depression, and reduced self-esteem, even after making adjustments for confounding variables such as socio-economic status, age, and comorbidities. More importantly, these relationships have appeared to be graded, as more severe forms of incontinence have demonstrated better linkages with depressive symptoms. Recent cohort evidence has also highlighted the overall clinical impact of this burden. In a few specific populations, such as women in the postpartum period, moderate to severe depression has been reported among nearly one-fifth of women experiencing urinary incontinence; the severity of their symptoms predicted depressive outcomes.

### 2.2. Anxiety, Distress, and Quality of Life

Looking beyond depressive symptomatology, SUI has also been strongly linked with broader dimensions of psychological distress, which include reduced self-esteem, anxiety, and impaired quality of life. Recent observational and cross-sectional studies have demonstrated that women who have SUI have reported alarmingly higher levels of psychological distress and anxiety compared to those without the condition of incontinence [Referred to Table 1]. This clearly indicates that the burden of this condition has extended throughout multiple domains of mental health [12]. It should be noted that symptoms of anxiety are not only secondary outcomes; they are closely intertwined with the lived experiences of the unpredictability of symptoms and social embarrassment.

Impairments in quality of life are specifically pronounced in occupational, social, and physical domains. The prevalence of SUI is highest among women in the 55–59 age range. Large population-based studies have shown that SUI is related to reduced mental well-being, social participation, and work productivity; this reflects the pervasive impacts of this condition on daily functioning [14]. Such effects are also compounded by underreporting and stigma, which might also delay the process of seeking help, and thereby increase the exposure to psychological distress. It should be further noted that a growing body of evidence has suggested that subjective symptom experience instead of objective clinical severity has been more strongly linked with emotional distress and anxiety. This kind of distinction has underscored the important role of the psychosocial context and perception in shaping the outcomes of mental health in SUI populations. This has reinforced the requirement for an integrative model which can move beyond biomedical interpretations of the condition.

### 2.3. Symptom Severity vs. Perceived Burden

A critical difference in the clinical understanding of SUI lies in the divergence between subjective perceived burden and objective symptom severity. While clinical measures such as volume of leakage and frequency can provide quantifiable indicators of disease severity, accumulating evidence has suggested that such metrics might not predict the psychological impact or health-related quality of life consistently [15]. Instead, the subjective experience of bother has emerged as a much more reliable determinant of stress and impairments in functionality.

Population-based studies have demonstrated that a few urinary symptoms such as mixed incontinence and stress are disproportionately perceived as being highly disturbing, irrespective of the measured severity. This indicates that the type of symptom and contextual interpretation might outweigh the merely clinical indices. In a similar way, research that has examined patient perceptions has highlighted that expectations, individual beliefs, and attitudes towards incontinence can shape the perceived impact of the condition significantly, thereby impacting satisfaction and decision-making during treatment [16]. More evidence has suggested that perceived burden has played an integral role in shaping behavioural outcomes, which include seeking help. Studies have also demonstrated that women have been more likely to seek medical care on the basis of how distressing or disruptive the symptoms are, instead of their clinical severity [17]. This kind of disconnect has personified the limitations of a pure biomedical framework, and has also reinforced the significance of integrating subjective experiences into both theoretical frameworks and the clinical assessment of SUI-related psychological outcomes.

From a collective standpoint, these findings have challenged purely symptom-centred biomedical interpretations of SUI. The weak correlation between psychological impairment and objective leakage severity has suggested that emotional distress can be shaped not just by physiological dysfunction, but also by perceived social threat, cognitive appraisal, and individual stress responsivity. This distinction is specifically crucial, as it can support the broader hypothesis that SUI might function as a chronic psychosocial stressor instead of a solely peripheral mechanical condition.

### 2.4. Psychological Outcomes Following Treatment

An exhaustive body of recent clinical research suggests that the treatment of SUI specifically through surgical interventions can be related to notable improvements in psychological outcomes. Prospective research which evaluated mid-urethral sling procedures has consistently reported reductions in both depressive and anxiety symptoms after the successful restoration of continence. As an example, longitudinal evidence has demonstrated that improvements in urinary symptoms can be correlated strongly with reductions in depression scores, which suggests a rather close relationship between psychological recovery and symptom resolution.

In a similar way, more recent studies of intervention have confirmed that the successful treatment of SUI has been related to notable improvements in mental health domains, which include depression, anxiety, and well-being. It should be noted that psychological distress might appear to mirror subjective improvements in urinary symptoms; this indicates that the recovery perceived by patients can play an important role in emotional outcomes. Such findings can support the belief that SUI-related psychological burden is, in part, reversible if the fundamental condition is managed effectively [18]. However, the outcomes of the treatment are not always uniformly positive across all types of individuals. Many patients do experience considerable psychological benefits, but there is variability in responses, which indicates that there are additional factors, such as coping mechanisms, mental health status, and psychosocial context, which might have an influence on trajectories of recovery. Emerging qualitative evidence has also highlighted that some individuals have continued to experience functional challenges or residual emotional challenges after treatment.

### 2.5. Persistent Affective Symptoms

While SUI treatment is frequently linked to improvements in psychological outcomes, a consistent finding from recent research shows that a critical subset of patients has continued to experience persistent affective symptoms after successful intervention. The evidence has shown that even though rates of anxiety and depression decline during the post-operative time period, residual symptoms continue to be a problem among a certain population of patients after the restoration of continence. For example, longitudinal data have indicated that nearly 8–10% of individuals have continued to meet the criteria for anxiety or depression symptoms a year after mid-urethral sling surgery, even after experiencing thorough improvements in urinary function [19].

This persistence suggests that psychological distress in SUI is not solely the result of ongoing physical symptoms. Instead, it might reflect much more complicated interactions between pre-existing vulnerability, chronic stress exposure, and sustained alterations in stress regulatory systems. In support of this interpretation, large-scale epidemiological evaluations have demonstrated that urinary incontinence is incorporated within the broader network of comorbidity, where conditions of depression can be impacted by cumulative socio-economic health and psychosocial factors [20]. In addition, emerging evidence has shown that depressive disorders and lower urinary tract symptoms have often coexisted through neurobiological mechanisms and shared psychological mechanisms, which indicates that psychological vulnerability might persist independently of symptom resolution.

The persistence of affective symptoms despite the successful restoration of continence might indicate the involvement of mechanisms which extend beyond ongoing peripheral dysfunction alone. Recurrent exposure to anticipatory anxiety, perceived loss of control, and social evaluative threat might also contribute the sustained activity of stress response systems, specifically neuroendocrine pathways related to HPA axis regulation. From this standpoint, the psychological burden observed in SUI might reflect broader biological adaptation related to stress instead of solely reactive emotional distress.

## 3. SUI as a Chronic Psychosocial Stressor

### 3.1. Characteristics of Chronic Stress in SUI

SUI can be conceptualised as a chronic psychosocial stressor because of its recurrent, persistent, and context-dependent nature; this is different from acute stress, which is more stimulus-bound and transient. Stress related to SUI can be characterised by consistent exposure to situations which can trigger the symptoms, and these triggers can be embedded within the everyday activities of the individual. Episodes of leakage can be completely unpredictable and thereby socially salient, which creates a consistent state of anticipatory concern and vigilance; this type of pattern aligns with established models relating to chronic stress. Considerable but repeated stressors can accumulate over a period of time for sustained physiological and psychological effects.

Experimental evidence has supported the overall biological relevance of this type of stress exposure. Animal model studies have demonstrated that chronic psychological stress can have a direct impact on lower urinary tract function through autonomic and new endocrine pathways, which suggests bidirectional interactions between bladder physiology and stress systems [21]. From a clinical standpoint, observational research has indicated that factors related to SUI, such as comorbidities, age, and lifestyle variables, can easily coexist within the broader vulnerabilities related to stress, which reinforces the multifaceted characteristics of this condition. In addition to this, severity-based analysis has shown that an increased burden of symptoms is also linked with more functional limitations and psychosocial strain, which indicates an overall cumulative stress effect over a period of time [22].

### 3.2. Anticipatory Anxiety and Social Evaluative Threat

Beyond physical symptom burden, SUI may also function as a chronic source of social evaluative stress. An important feature of SUI as a chronic stressor is the presence of anticipatory anxiety, which is moderated by the consistent expectation of symptom occurrence in socially sensitive contexts. Women who have SUI have frequently reported a continuous heightened state of vigilance, where routine activities such as exercising, walking, or even engaging in social interactions can be accompanied by an unnerving concern about a potential leakage [23]. This kind of anticipatory element can transform SUI from a mere episodic condition into a continuous psychological burden, since the fear of future events can be extremely impactful, almost as much as the symptoms themselves.

Qualitative evidence has highlighted that this phenomenon can be referred to as a state of constant worry, where individuals describe ongoing mental preoccupation with maintaining control in public settings and avoiding embarrassment. This has aligned closely with the definition of social evaluative threat, where stress responses can be magnified by the overall perceived risks of humiliation or negative judgement. Observational studies have demonstrated that SUI can be linked significantly with higher anxiety levels, thereby reinforcing the role of psychological anticipation in shaping an innate feeling of distress [24].

In response, individuals have often adopted coping behaviours or compensatory behaviours such as fluid restriction, pre-emptive voiding, or the complete avoidance of specific environments to mitigate the perceived risks [25]. These strategies do provide short-term control, but they also reinforce anxiety even more as the individual must maintain a heightened perception of threat. From a collective standpoint, social evaluative threat and anticipatory anxiety can be the core mechanisms through which SUI contributes towards this sustained state of psychological stress. Persistent anticipatory anxiety and the perceived loss of control may contribute towards the repeated activation of stress response systems associated with HPA axis regulation.

### 3.3. Behavioural Avoidance and Stress Reinforcement

Behavioural avoidance has represented an important mechanism through which SUI has contributed to the magnification and sustenance of chronic psychological stress. Individuals have frequently restricted or modified their daily activities, such as social participation, exercise, or travel, to minimise the risks of leakage and related embarrassment. Evidence has also shown that women with stress urinary incontinence show less favourable health-related lifestyle patterns in comparison to those without the condition; this suggests that the burden of symptoms might influence everyday functioning and behavioural adaptation [26]. Over time, sustained hypervigilance and chronic stress anticipation may reinforce prolonged neuroendocrine stress activation and contribute to increased allostatic load.

Emerging real-time data has further demonstrated that activity avoidance can be linked dynamically to the anticipation of symptoms as individuals have reduced engagement in routine behaviours as a response to perceived risks [27]. While these kinds of strategies might offer control in the short term, they might also reinforce anxiety by sustaining the cognition of threat and by limiting exposure to corrective experiences.

From a physiological standpoint, chronic psychological stress has been linked with adverse behaviours, which might also exacerbate bladder dysfunction since experimental models have shown stress-induced increases in terms of neural activity and bladder sensitivity [28]. Altogether, data have painted a picture which shows a clear feedback loop in which behaviours of avoidance have perpetuated both symptom severity and psychological distress.

These stress-related behavioural responses might manifest through measurable psychosocial indicators, which include the avoidance of physical activity, reduced social participation, sleep disturbance, occupational disruption, sustained anxiety regarding leakage unpredictability, and heightened symptom vigilance. Such patterns are consistent with chronic stress adaptation instead of isolated episodic embarrassment.

### 3.4. Comparison with Other Chronic Stress Conditions

The psychosocial profile of SUI shares a few important features with other types of chronic stress-related conditions, specifically in its nature of persistent impact on the emotional well-being and the regular functioning of the patient. Similar to chronic illnesses such as chronic pain or other types of long-term health disorders, SUI has been linked with sustained increases in anxiety, stress, and other depressive symptoms, and this reflects an ongoing dialogue between psychological response and psychological dysfunction. Population-based studies have indicated that urinary incontinence can be linked consistently with elevated levels of reduced self-esteem and stress, even after health-related and demographic variables are controlled for.

More importantly, the bidirectional link which is observed between lower urinary tract symptoms and mental health disorders mirrors the patterns which are observed in other types of chronic stress conditions, where psychological distress can contribute to and also result from the processes of these diseases. In addition to this, research on disorders of the pelvic floor has demonstrated more broadly the comparable influence on the quality of life and emotional well-being of women, thereby further placing SUI in a wider spectrum of conditions related to stress [29].

From a collective standpoint, these psychological characteristics have positioned SUI as more than just a peripheral urogynecological disorder, but also as a persistent chronic stress exposure which is capable of engaging the central stress regulatory systems. Repeated experiences of anticipatory anxiety, social evaluative threat, behavioural vigilance, and perceived loss of control might contribute towards the sustained activation of neuroendocrine stress pathways, specifically the hypothalamic–pituitary–adrenal (HPA) axis. Through prolonged cortisol signalling and stress sensitisation, these mechanisms can provide a biologically plausible pathway which links chronic symptom-related stress with psychological dysfunction and vulnerability. The following section thereby examines the neuroendocrine mechanisms underlying the regulation of chronic stress.

From a clinical perspective, this chronic stress framework might potentially be reflected through measurable indicators, which include anxiety severity, perceived stress burden, cortisol dysregulation, sleep disturbance, health-related quality of life impairment, and behavioural avoidance patterns.

## 4. HPA Axis Dysregulation in Chronic Stress

### 4.1. Physiology of the HPA Axis

The hypothalamic–pituitary–adrenal (HPA) axis is the integral neuroendocrine system which regulates physiological responses to stress. The activation starts in the hypothalamus with the release of the corticotropin-releasing hormone (CRH); this stimulates the anterior pituitary gland to secrete the adrenocorticotropic hormone (ACTH). This ACTH then triggers the adrenal cortex to release cortisol, thereby facilitating immune, metabolic, and behavioural adaptations to stress. Cortisol exerts its effects through the glucocorticoid receptors.

Cortisol also exerts its effects through glucocorticoid receptors, which are distributed throughout the body and brain, specifically in regions such as the hippocampus, where it can modulate stress feedback mechanisms. Under normal circumstances, the HPA axis is regulated strictly through negative feedback; this ensures a transient form of activation. However, integrative models have emphasised that dysregulation might occur when this kind of feedback system is disrupted, thereby altering homeostasis and stress responsivity [30].

### 4.2. Cortisol Dynamics and Stress Response

Cortisol is known as the primary effector hormone of the HPA axis and it can play a central role in coordinating the physiological response towards stress. Acute stress can trigger a fast surge in the secretion of cortisol, thereby facilitating the mobilisation of energy, adaptive behavioural responses, and modulation of immune functions [31]. Under idealised situations, the release of cortisol can follow a strictly regulated circadian rhythm and it is also governed by negative feedback mechanisms, which ensure that recovery is timely after exposure to stress. However, chronic stress can alter these dynamics. Biological and mathematical models can demonstrate the continuous activation of the HPA axis; this can lead to continuous cortisol dysregulation on timescales that extend beyond the immediate exposure to stress, which may result in a sustained hormonal imbalance [32]. This kind of dysregulation can be related to impaired sensitivity to feedback and it has also been linked to a host of neurological and psychological disorders, which include cognitive dysfunction and depression [33].

### 4.3. Allostatic Load and Chronic Activation

Allostatic load refers to the cumulative physiological burden which can be imposed by the chronic or repeated activation of various stress response systems, specifically the HPA axis. Instead of reflecting adaptive regulation, the persistent activation of stress response systems can contribute to cumulative physiological burden throughout neuroendocrine, metabolic, and immune pathways; this process is conceptualised as allostatic overload [34]. Over a period of time, this type of dysregulation can reflect a clear shift from effective stress adaptation towards maladaptive functioning. Empirical evidence has also demonstrated that heightened allostatic load is closely related to adverse mental health outcomes, which include anxiety and depression, along with broader patterns of physiological dysregulation [35]. In addition to this, clinical studies have also indicated that individuals with depressive disorders have exhibited higher allostatic loads, which are accompanied by metabolic disturbances, highlighting the systemic results of exposure to chronic stress [36].

### 4.4. Stress Sensitisation and Affective Vulnerability

Sensitisation to stress can be referred to as the process by which early life exposure to stress can lower the threshold for future responses to stress; this increases vulnerability to affective disorders. Evidence has indicated that this type of HPA axis regulation can play an integral role in this process, as prior stress exposure can lead to prolonged or exaggerated physiological responses to later stressors [37]. This type of heightened reactivity can contribute to the development of vulnerability, even when there is an absence of ongoing stress. Conditions of chronic stress can also reinforce this kind of sensitisation. Studies have revealed that the persistent activation of the stress response dynamic, which includes both autonomic and HPA axis pathways, can be related to the elevated susceptibility to mood disturbances, pain, and emotional dysregulation [38]. Collectively, these findings indicate that repeated exposure to stress can recalibrate stress systems, thereby predisposing individuals towards a sustained form of affective vulnerability. Clinically, persistent HPA axis activation might help explain the heightened stress sensitivity, sustained anxiety, and prolonged affective symptoms observed in a subset of patients with SUI.

The persistent deregulation of stress response systems might further influence downstream molecular pathways, which include epigenetic regulation, glucocorticoid receptor signalling, inflammatory activity, and neuroplasticity, thereby linking exposure to chronic stress with longer-term biological vulnerability.

## 5. Molecular and Epigenetic Mechanisms in Stress-Related Disorders

### 5.1. Epigenetic Regulation and Environmental Stress

Building on chronic HPA axis activation and cortisol dysregulation, epigenetic mechanisms might represent one pathway through which prolonged stress exposure might become biologically embedded over time. The persistent dysregulation of stress response systems might not remain limited only to neuroendocrine activation, but may also produce epigenetic alterations and longer-term molecular alterations which are related to stress-related psychopathology [39].

Epigenetic regulation can provide an important mechanism through which environmental stressors exert long-term effects on the expression of genes, without making alterations in the DNA sequence. Processes such as histone modification, DNA methylation, and non-coding RNA activity can enable exposure to stress, producing sustained changes in physiological and neural functioning [40]. More importantly, stress-related alterations in genes involved in neuroplasticity, including brain-derived neurotrophic factor (BDNF), have also been implicated in the development of various affective disorders [41]. These mechanisms can emphasise how chronic stress can quickly become biologically incorporated, thereby contributing to consistent vulnerability towards psychological dysfunction.

### 5.2. Glucocorticoid Signalling: NR3C1

Glucocorticoid signalling is integral to the regulation of stress and it is mediated mostly by the glucocorticoid receptors, which are encoded by the NR3C1 gene. This receptor can play an important role in HPA axis feedback, thereby modulating cortisol responses and maintaining homeostasis. Epigenetic alterations, specifically the DNA methylation of NR3C1, have been linked with disrupted responsivity towards stress and elevated vulnerability towards disorders relating to stress [42]. Genome-wide studies have also demonstrated that dysregulated NR3C1 expression can be linked to altered cortico-limbic function, thereby reinforcing its function in stress adaptation and emotional regulation [43].

### 5.3. FKBP5 and Stress Reactivity

FKBP5 is a core regulator of glucocorticoid receptor sensitivity and it plays a crucial role in the modulation of stress reactivity in the HPA axis. An increase in the expression of FKBP5 can reduce the affinity of the receptor for cortisol; this can impair negative feedback while prolonging stress responses. Epigenetic modifications, specifically stress-induced methylation alterations of DNA, have been associated with altered responsivity towards stress and increased susceptibility towards metabolic and psychiatric disorders [44]. These types of alterations can also contribute to dysregulated stress signalling and thereby are implicated in the overall development of central nervous system disorders. Even though currently exploratory, stress-related epigenetic profiles might eventually contribute towards the identification of individuals who have increased vulnerability towards chronic psychological distress and maladaptive stress responses.

## 6. Neuroplasticity and Inflammatory Pathways

### 6.1. BDNF and Neuroplasticity in Depression

BDNF has been a crucial regulator of neuroplasticity, which influences synaptic connectivity, neuronal survival, and adaptive brain function. In depression, the reduced expression of BDNF has been consistently linked to impaired neuroplastic processes, specifically within the prefrontal cortex and hippocampus, regions which are integral to mood regulation [45]. Chronic stress has been an important factor which has contributed to this kind of reduction and has been linked to environmental exposure and functional and structural changes in the brain [Referred to Table 2]. Integrative models of depression can also emphasise that disrupted neuroplasticity can be a reason for emotional and cognitive dysfunction; BDNF can play a central mediating role in this process [46]. These findings have demonstrated that neuroplasticity is an important biological substrate in affective disorders related to stress. Within the proposed framework, these neuroplastic alterations are considered relevant primarily to the extent that chronic stress exposure in SUI may contribute towards sustained affective vulnerability.

### 6.2. Inflammation and Stress Signalling

Chronic stress has been increasingly recognised in inflammatory pathway activation, which can interact closely with the endocrine and neural stress systems. Evidence has shown that prolonged psychosocial stress induces immune responses in specific regions, thereby altering cytokine activity and also contributing towards changes in gut–brain signalling and neuroplasticity [48]. These kinds of inflammatory processes are not peripheral phenomena solely, but they are closely linked to the functions of the central nervous system. Experimental studies have also shown that oxidative stress, neuroinflammation, and apoptotic pathways are increased under conditions of chronic stress and are linked with depression [49]. Altogether, it can be said that these findings have highlighted inflammation as an important mediator which links exposure to stress to affective disorders and neural dysfunction. These inflammatory mechanisms may hold relevance for understanding persistent mood-related symptoms and stress-related somatic burden in chronic SUI populations.

### 6.3. Integrated Biological Pathways

Inflammatory processes and neuroplasticity cannot operate in isolation; instead, they develop interconnected biological pathways that can underpin disorders related to stress. Chronic stress can induce coordinated alterations throughout immune, neural, and molecular systems, where inflammatory signalling can impair neuroplasticity and also disrupt mechanisms of neuronal recovery [50]. BDNF is central to this integration as it links cellular resilience with exposure to environmental stress; this is modulated by both neuroendocrine and inflammatory processes. These kinds of interactions have a systems-level framework where inflammation, stress, and neuroplasticity have converged, thereby contributing towards the persistence and development of depressive pathology. Impaired neuroplastic adaptation might also lead to a potential explanation for persistent affective vulnerability despite successful improvements in urinary symptoms.

Within the proposed framework, chronic psychosocial stress can initially activate HPA axis pathways and also dysregulate cortisol signalling. Sustained glucocorticoid exposure might subsequently alter stress-responsive epigenetic regulators such as NR3C1 and FKBP5, which in turn can influence neuroplasticity-related pathways and inflammatory signalling, including BDNF activity [51]. These interacting mechanisms might provide a biologically viable cascade which links chronic stress exposure with affective vulnerability.

## 7. Integrating SUI into a Translational Psychiatry Framework

The cumulative evidence in relation to psychosocial stress, epigenetic modification, and neuroendocrine dysregulation has supported the integration of SUI into a broader translational psychiatry framework.

### 7.1. From Peripheral Disorder to Central Stress Processing

Fundamentally, urinary incontinence has been understood as a peripheral mechanical disorder which primarily involves pelvic floor function and urethral support. The cumulative evidence has supported a basic reconceptualisation of SUI as a condition with stress-processing implications, which can be central to this new understanding. The unpredictable, chronic, and evaluative nature of experiences related to SUI has positioned it as a consistent psychosocial stressor that is capable of engaging systems which regulate stress. The repeated activation of stress pathways, such as the HPA axis, along with procedures such as behavioural avoidance, anticipatory anxiety, and perceived loss of control, has suggested that SUI might contribute towards sustained affective and neuroendocrine dysregulation. More importantly, the persistence of psychological symptoms after successful physical treatment has also supported the belief that the central mechanisms, instead of peripheral dysfunction, can play the most critical role in shaping outcomes in the long term.

### 7.2. Biopsychosocial Integration Model

A biopsychosocial integration model can provide an exhaustive framework for comprehending SUI as a multi-dimensional condition which arises from the dialogue between psychological, biological, and social factors. From a biological standpoint, SUI can involve dysfunction in the pelvic floor, alongside the dysregulation of stress-responsive systems, which include associated neuroendocrine pathways and the HPA axis. Psychologically, factors such as perceived loss of control, anticipatory anxiety, and maladaptive coping behaviours can contribute to sustained distress. Socially, embarrassment, stigma and restriction on activity can also reinforce this kind of burden. More importantly, these cannot operate independently, but they do interact dynamically, thereby creating feedback loops that can sustain both psychological vulnerability and symptom perception. The integrative model has highlighted SUI as a condition which is shaped by the consistent interplay between central stress processing and peripheral dysfunction. These interactions are likely to operate through reciprocal feedback loops, whereby distress related to symptoms increases behavioural vigilance, anticipatory anxiety, and stress system activation, which can subsequently intensify symptom perception, maladaptive coping behaviours, and emotional burden over time.

### 7.3. Vulnerability vs. Resilience Profiles

The heterogeneity of psychological outcomes in SUI has suggested the presence of resilience profiles and distinct vulnerability among affected individuals. While some patients have experienced persistent and significant affective symptoms, others have demonstrated psychological stability and adaptive coping even after having symptoms of comparable severity. This kind of variability has indicated that individual differences in stress responsivity, biological sensitivity, and coping strategies can play an important role in shaping outcomes. From a biological standpoint, the variations in HPA axis regulation and stress sensitisation might predispose individuals towards increased affective vulnerability. Psychologically, maladaptive behaviours such as heightened threat perception and avoidance can also reinforce this kind of vulnerability. On the other hand, resilience might be supported by lower perceived burden and effective coping mechanisms, thereby highlighting the significance of differences on an individual level in the proposed framework.

### 7.4. Proposed Stress-Epigenetic Model

Building on previous evidence, this p erspective proposes a stress-epigenetic model where SUI acts as a chronic psychosocial stressor which has the capability of inducing sustained biological alterations. In the early stages, the unpredictability of symptoms and social evaluative threat might primarily generate acute psychological distress and greater vigilance. With consistent exposure over time, the repeated activation of stress response systems might contribute to increased allostatic load, chronic HPA axis dysregulation, and longer-term biological adaptation. The repeated activation of stress pathways, specifically the HPA axis, might lead to dysregulated dynamics of cortisol and, thereby, increased allostatic load. Over a period of time, this chronic neuroendocrine activation might be biologically incorporated through epigenetic mechanisms, which include the altered DNA methylation of stress-responsive genes such as FKBP5 and NR3C1 [52]. These kinds of molecular changes might also have an influence on pathways of inflammatory signalling and neuroplasticity, thereby contributing towards persistent vulnerability. Importantly, direct epigenetic evidence, which is specifically derived from SUI populations, has remained limited, and the proposed framework therefore needs to be interpreted as a biologically viable and theoretically informed model instead of an empirically established causal pathway.

From a collective standpoint, these biological mechanisms might hold potential translational relevance beyond theoretical interpretations alone. The dysregulation of stress response systems, inflammatory activation, stress-related epigenetic alteration, and impaired neuroplastic adaptation might help explain why individuals experience persistent psychological distress even after comparable symptom severity or successful continence treatment [53]. From a clinical perspective, these pathways might thereby contribute towards future models of biomarker-informed assessment, psychological risk stratification, and interdisciplinary intervention strategies in SUI populations.

Nevertheless, direct longitudinal translational evidence which integrates psychological assessment, epigenetic, and neuroendocrine profiling in SUI populations has remained limited, and the proposed work therefore needs to be interpreted as a conceptual translational model which requires further empirical validation.

The proposed model has therefore offered a conceptual framework for understanding how chronic symptom-related stress in SUI might interact with epigenetic, neuroendocrine, neuroplastic, and inflammatory mechanisms related to affective vulnerability.

## 8. Translational and Clinical Implications

### 8.1. Risk Stratification and Screening

The effective management of SUI may require the early recognition of individuals who are at high risk of both psychological and physical complications. A growing body of evidence has suggested that depression and SUI can share common risk profiles, which include factors such as comorbid conditions, poor self-reported health, and lifestyle variables, thereby enabling the identification of high-risk groups for the purpose of targeted screening. In addition to this, systematic evidence has highlighted the significance of integrating mental health assessment into the routine assessment of urinary incontinence, due to the strong linkages between depressive outcomes and SUI. The screening approaches should thereby extend beyond the detection of symptoms to include validated tools for assessing psychological distress, as this can facilitate early interventions and thereby improve patient outcomes.

Patients who present with severe psychosocial distress, behavioural avoidance, anticipatory anxiety, persistent affective symptoms, or sleep disturbance might benefit from routine psychological screening along with conventional urogynecological assessment [54]. Standardised instruments assessing anxiety severity, perceived stress, depressive symptomatology, and health-related quality of life might help in identifying individuals at elevated risk of chronic psychological burden.

### 8.2. Role of Psychological and Biological Markers

The integration of biological and psychological markers can offer a promising approach for understanding individual variability in the outcomes of SUI. Psychological markers, which include anxiety, perceived symptom burden, and coping style, can predict treatment response and quality of life in a more effective manner than objective symptom severity. Concurrently, biological markers such as epigenetic modifications and cortisol in stress-related genes have been recognised increasingly as indicators of effective vulnerability and stress system involvement [55]. Collectively, these markers can provide a multi-dimensional assessment framework, enabling the more in-depth identification of individuals who are at risk, as well as personalised management strategies. Within this proposed framework, biological mechanisms are not interpreted as isolated molecular abnormalities, but rather as potential contributors to clinical heterogeneity in psychological outcomes. Dysregulated cortisol signalling, impaired neuroplastic adaptation, and altered stress responsivity might collectively assist in explaining why some individuals demonstrate persistent affective symptoms despite the successful mechanical treatment of SUI [56]. Even though these biomarkers are not used routinely in clinical practice, they might provide a basis for further personalised psychological risk assessment along with longitudinal monitoring strategies.

Future translational approaches can also incorporate longitudinal assessments of inflammatory markers, cortisol regulation, and stress-related epigenetic profiles to improve the identification of patients who are vulnerable to persistent affective dysregulation.

### 8.3. Interdisciplinary Management Strategies

Due to the multifaceted nature of SUI, effective management may require an interdisciplinary strategy which can integrate psychological, urogynecological, and behavioural interventions. Conventional treatments, surgical procedures, and pelvic floor muscle training might address the mechanical facets of SUI, but evidence has also indicated that the incorporation of psychological support can improve outcomes, specifically in patients who have elevated distress levels and maladaptive patterns of coping [57]. Behavioural interventions which target avoidance and anxiety, along with counselling and patient education, can also assist in reducing perceived burden and thereby improve adherence to treatment [58]. This kind of integrated strategy recognises both the central and peripheral components of SUI. Clinically, the latest framework might support more integrated management models where pelvic floor rehabilitation is combined with stress management strategies, psychological assessment, and monitoring of persistent affective symptoms in high-risk patients [59]. Patients who demonstrate a significant psychosocial burden might benefit from referral for cognitive behavioural therapy, behavioural stress reduction strategies, or multidisciplinary pelvic health services which integrate mental health support and urogynecology.

Such approaches may help shift SUI management from a purely symptom correction model towards a broader biopsychosocial framework incorporating the prevention of chronic stress adaptation and long-term psychological vulnerability.

## 9. Limitations and Conceptual Boundaries

This perspective is conceptual and integrative in nature and therefore does not include an original clinical biomarker assessment of HPA axis activity, cortisol rhythms, inflammatory markers, or epigenetic profiles in SUI populations. The dependence on heterogeneous evidence from experimental, clinical, and molecular studies has introduced variability in its interpretation. Crucially, direct empirical evidence specifically linking SUI with stress-related epigenetic modification, inflammatory alteration, HPA axis dysregulation, or neuroplastic changes remains limited. Most of the proposed framework is thereby extrapolated from the broader psychiatric, epigenetic, and chronic stress literature instead of evidence derived directly from biomarker studies, which are conducted in SUI populations. In addition to this, empirical studies which have specifically linked SUI to epigenetic modifications also remain limited, thereby constraining assured conclusions. From a conceptual standpoint, the proposed model may oversimplify complicated bidirectional interactions. These boundaries have also highlighted the requirement for multi-level and longitudinal research for refining and validating the framework. As a result, the proposed framework needs to be interpreted as a theoretically informed and biologically viable conceptual model instead of a definitive causal explanation of psychological outcomes related to SUI.

## 10. Future Research Directions

Future research should prioritise multilevel longitudinal studies to empirically evaluate the proposed stress-epigenetic framework in SUI. Prospective cohort designs integrating psychological assessment, repeated neuroendocrine measurements, epigenetic profiling, and inflammatory biomarkers may help clarify temporal and potentially causal relationships. Such studies should also examine individual variability through vulnerability-resilience models that incorporate environmental, behavioural, and genetic moderators. Experimental and intervention research should evaluate whether psychological or behavioural treatments can modify biological stress markers. Interdisciplinary work bridging psychiatry and urogynecology will be essential for developing mechanism-informed screening and integrated management strategies.

## 11. Conclusions

This perspective proposes a conceptual stress-epigenetic framework through which SUI might be understood not solely as a peripheral mechanical disorder but also as a condition which has broader neurobiological and psychosocial implications. Through the integration of evidence from neuroendocrine regulation, chronic stress physiology, inflammation, epigenetics, and neuroplasticity, this perspective highlights the potential relevance of translational psychiatry perspectives in comprehending persistent affective vulnerability in SUI. Even though direct molecular evidence in SUI populations is limited, the framework has offered a biologically viable basis for interdisciplinary research in the future and more integrated strategies towards specialist management.

## Figures and Tables

**Table 1:** Epidemiology table (source: created by author).

Outcome	Reported Association with SUI
Depression	Increased prevalence [13]
Anxiety	Elevated symptom burden
QoL impairment	Reduced physical/social functioning
Behavioural avoidance	Frequently reported

**Table 2:** Summary of key stress-related epigenetic mechanisms relevant to the proposed framework (source: created by author).

Gene/Pathway	Epigenetic Modification	Proposed Functional Effect	Type of Evidence
**NR3C1**	Increased DNA methylation of glucocorticoid receptor-related regions	Reduced glucocorticoid receptor sensitivity and impaired HPA axis feedback regulation	Human stress-related psychiatric studies and epigenetic research [47]
**FKBP5**	Stress-associated alterations in DNA methylation	Dysregulated glucocorticoid signalling and prolonged stress responsivity	Psychiatric epigenetic studies and chronic stress models
**BDNF-related pathways**	Stress-associated epigenetic suppression of BDNF expression	Impaired neuroplasticity and affective vulnerability	Neurobiological and depression-related studies

## Data Availability

No new data were created or analyzed in this study. Data sharing is not applicable to this article.
